# Uniaxially Strained Graphene: Structural Characteristics and G-Mode Splitting

**DOI:** 10.3390/ma15010067

**Published:** 2021-12-22

**Authors:** George Kalosakas, Nektarios N. Lathiotakis, Konstantinos Papagelis

**Affiliations:** 1 Department of Materials Science, University of Patras, GR-26504 Rio, Greece; 2 Theoretical and Physical Chemistry Institute, National Hellenic Research Foundation, Vass. Constantinou 48, GR-11634 Athens, Greece; lathiot@eie.gr; 3 Department of Solid State Physics, School of Physics, Aristotle University of Thessaloniki, GR-54124 Thessaloniki, Greece; kpapag@physics.auth.gr

**Keywords:** graphene, uniaxial strain, bond lengths, bond angles, G-band splitting, density functional theory, molecular dynamics

## Abstract

The potential use of graphene in various strain engineering applications requires an accurate characterization of its properties when the material is under different mechanical loads. In this work, we present the strain dependence of the geometrical characteristics at the atomic level and the Raman active G-band evolution in a uniaxially strained graphene monolayer, using density functional theory methods as well as molecular dynamics atomistic simulations for strains that extend up to the structural failure. The bond length and bond angle variations with strain, applied either along the zigzag or along the armchair direction, are discussed and analytical relations describing this dependence are provided. The G-mode splitting with strain, as obtained by first principles’ methods, is also presented. While for small strains, up to around 1%, the G-band splitting is symmetrical in the two perpendicular directions of tension considered here, this is no longer the case for larger values of strains where the splitting appears to be larger for strains along the zigzag direction. Further, a crossing is observed between the lower frequency split G-mode component and the out-of-plane optical mode at the Γ point for large uniaxial strains (>20%) along the zigzag direction.

## 1. Introduction

Graphene belongs to the family of atomically thick two-dimensional materials, exhibiting extraordinary electronic, optical, and mechanical properties [1]. Due to its enormous mechanical stretchability and strength, graphene provides a platform for the implementation of the strain engineering concept, namely, the tuning of its intrinsic properties by inducing controlled mechanical macroscopic or microscopic strain fields, e.g., uniaxial or biaxial [2,3]. Since pristine graphene exhibits gapless electronic band structure, strain engineering has been utilized to open the gap and to induce giant pseudomagnetic fields [4]. A combination of shear and uniaxial deformation between 12% and 17% can open a gap from 0 to 0.9 eV [5], while highly strained nanobubbles that form when graphene is grown on top of platinum, present pseudomagnetic fields higher than 300 T [6]. Strain-induced pseudo-magnetic fields greater than 300 Tesla appear in graphene nanobubbles. It has been also shown that a uniaxial strain either on the zigzag or on the armchair direction, can open a band gap in the phonon spectrum of graphene [7]. Further, the absorption and diffusion of hydrogen atoms on graphene is also affected by mechanical strain [8].

Many techniques have been employed to induce mechanical strain into graphene lattice, such as bending of flexible substrates, control wrinkling, and piezoelectric straining [2,9]. For example, by transferring graphene on top of uniaxially deformed elastomeric substrates and then releasing the strain, buckling induced wrinkles with a non-uniform mechanical strain field can be formed [2]. Furthermore, graphene can be subjected to tensile biaxial strain by warming up the underlaying substrate, due to the negative thermal expansion coefficient of graphene and the significant thermal strain mismatch with the substrate [10]. Besides, local strain engineering is a rapidly evolving technology enabling the next-generation of electronic and photonic devices [11].

Experimental observations indicate that graphene is the strongest material known, exhibiting an intrinsic strength of 42 N/m and a Young’s modulus equal to 340 N/m, corresponding to effective 3-dimensional values of 130 GPa and 1.0 TPa, respectively [12]. The mechanical response and the elastic properties of graphene under uniaxial tensile strain have been extensively studied theoretically [7,13,14,15,16,17,18,19,20,21,22]. These works, employing methods from first principles [7,19,20], atomistic simulations [15,17,18,20] and even analytical calculations [22], have successfully predicted Young’s moduli around the experimental value of 1 TPa. The measured value of the intrinsic strength has also been obtained by numerical computations [7,17,20]. The compressive response of graphene under uniaxial strain and the resulting buckling has been investigated too [23,24,25]. Apart from bulk graphene, the mechanical behavior of narrow-width graphene nanoribbons has also been examined theoretically [26,27,28,29,30,31].

From the early days of graphene research, Raman spectroscopy in tandem with cantilever-beam arrangements was utilized to examine the type (compressive versus tensile, biaxial or uniaxial) and the amount of mechanical strain in graphene samples. A great effort both experimentally and theoretically has been devoted to investigating the response of the main peaks of the Raman spectrum, and especially the doubly degenerate G-band, upon strain [32,33,34,35,36,37,38,39,40,41,42,43]. It was demonstrated that upon uniaxial tensile strain the G peak softens, and the degeneracy of G mode is lifted, splitting into two distinct components, the so-called G**^−^** and G**^+^** [44,45]. The split components possess mode eigenvectors parallel (G**^−^**) and perpendicular (G**^+^**) to the strain direction. The strain rate of softening for free-hanging graphene is about −18.6 and −36.4 cm^−1^/% for the G**^+^** and G**^−^** components, respectively [45]. Further, the shift rate of the G**^−^** and G**^+^** modes is found to be independent of the direction of strain for strains up to about 1% [45], while their relative intensities depend on light polarization, thus providing a useful tool to probe the graphene crystallographic orientation with respect to the strain axis [44,45].

Here we theoretically calculate the strain dependence of bond lengths and bond angles in monolayer graphene under uniaxial tensile strain up to the regime of structure failure. We examine graphene’s mechanical response using methods from first principles as well as atomistic molecular dynamics simulations. Results are obtained for uniaxial strains applied along either the armchair or the zigzag direction. The numerically obtained variation of the atomic level geometrical characteristics with strain is fitted to simple analytical relations for more efficient use in other studies. Further, the optical phonons at the center of the Brillouin zone (Γ point) are calculated through density functional theory in order to investigate the softening rate and the splitting of the doubly degenerate G band with uniaxial strain along the armchair or the zigzag direction.

The next section briefly presents the theoretical methods used in this work, the following section discusses our results, and the final section concludes our study.

## 2. Methods

Molecular dynamics (MD) simulations are performed by implementing the previously computed empirical force fields, especially designed for graphene using results from first principles’ methods, which have been derived through appropriate deformations of the structure [20,46,47]. Concerning uniaxial tensile strains, that are of interest here, only the in-plane bond stretching and angle bending potentials are used [20]. The mechanical response of graphene is obtained by applying constant forces to all atoms belonging to the proper edges of graphene. For strains along the armchair direction of graphene, the forces are applied to the atoms of the zigzag edges, while for strains along the zigzag direction the forces are applied to the atoms of the armchair edges. Opposite tensile forces are applied to the atoms of the opposite edges in each case. More details regarding the molecular dynamics simulations that lead to the equilibrium structure of graphene under tension can be found in reference [20]. For a particular value of force/strain, the bond lengths and bond angles of the uniaxially strained graphene are measured at the center of the structure to avoid edge effects.

**Figure 1:**
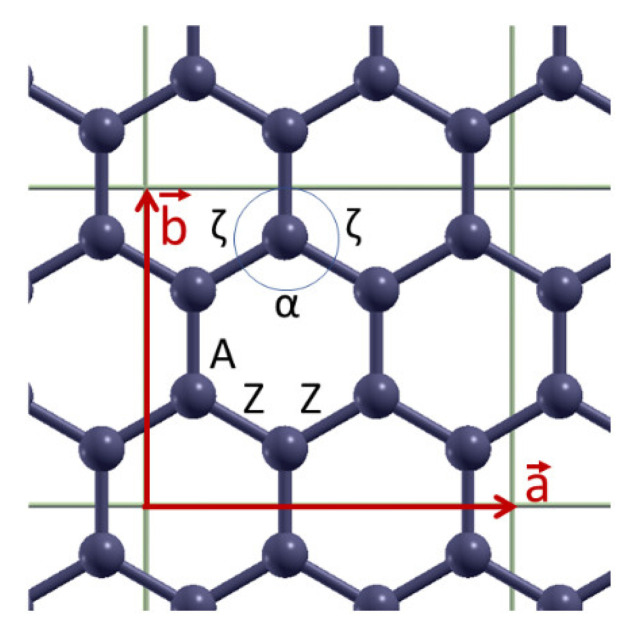
The rectangular 8-atom unit-cell considered for our DFT calculations, where bonds of A and Z type and angles of α and ζ type are shown. These bonds and angles are no longer equivalent for finite uniaxial strains along either zigzag (horizontal) or armchair (vertical) directions.

For density functional theory (DFT) calculations we used the Quantum Espresso suite [48,49,50]. We performed two sets of calculations with the functionals LDA/PZ [51] and GGA/PBE [52]. Regarding the dependence of the geometrical characteristics on strain we adopted an 8-atom rectangular unit-cell, shown in Figure 1, in order to eliminate possible finite-size effects on the obtained geometrical characteristics. For the perpendicular, out of plane direction we assumed a periodicity of 20 Å. We used the PAW pseudopotentials [53] from PSlibrary [54], with plane-wave cutoffs 50 and 400 Ryd for the wavefunction and density, respectively. Further, we used Gaussian type smearing and a value of the Gaussian spreading 0.02 Ryd, while for the reciprocal space a 24 × 24 × 1 sampling was considered.

After optimizing all structural parameters for both functionals, the strain was applied by manually extending the corresponding lattice dimension keeping it frozen and performing optimization of all atomic positions as well as the vertical to the strain unit cell dimension. For the phonon calculations, we also used the Quantum Espresso code with a primitive 2-atom unit cell and all other parameters being the same as above, apart from the k-points sampling that was 48 × 48 × 1 and the recommended tighter convergence criteria for phonon calculations. For the primitive unit cell, we also calculated the same geometrical characteristics we obtained for the 8-atom unit cell. The results for the two unit cells were almost the same, apart from tiny deviations in bond lengths for finite strain in the armchair direction when the bond crosses the primitive unit cell border.

## 3. Results and Discussion

In the first part, we present results regarding the deformation of bond lengths and bond angles upon uniaxial strain applied along the armchair or along the zigzag directions and in the second part we discuss the optical phonon frequencies at the Γ point, i.e., at the center of the Brillouin zone.

### 3.1. Bond Lengths and Bond Angles of Uniaxially Strained Graphene

**Figure 2:**
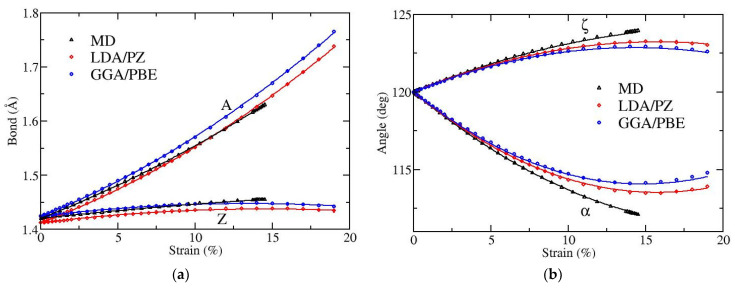
Strain dependence of (**a**) bond lengths and (**b**) bond angles, for uniaxial strain along the armchair direction of graphene. A and Z in (**a**) [α and ζ in (**b**)] correspond to the different kinds of bonds [angles], as shown in Figure 1. Black points show results obtained by MD, while red and blue points show data obtained by DFT using LDA and GGA functionals, respectively. Lines represent fits with quadratic polynomials [see Equations (1) and (2)].

**Figure 3:**
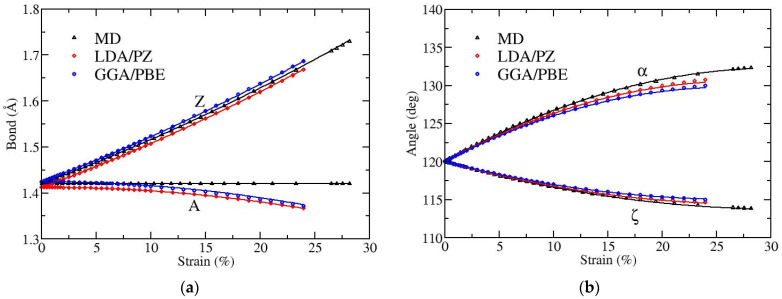
Strain dependence of (**a**) bond lengths and (**b**) bond angles, for uniaxial strain along the zigzag direction of graphene. A and Z in (**a**) [α and ζ in (**b**)] correspond to the different kinds of bonds [angles], as shown in Figure 1. Black points show results obtained by MD, while red and blue points show data obtained by DFT using LDA and GGA functionals, respectively. Lines represent fits with quadratic polynomials [see Equations (1) and (2)].

Figure 2 and Figure 3 depict the variation with strain of bond lengths [panels (a)] and bond angles [panels (b)] when the applied strain is along the armchair and along the zigzag directions, respectively. There are two distinct kinds of bonds (A and Z) as well as of angles (α and ζ), as illustrated in Figure 1. The angles α and ζ are not varied independently since they satisfy the condition α + 2ζ = 120°.

As expected, when the strain is along the armchair direction the lengths of both bonds A and Z increase, the angle α decreases while the angle ζ increases, as the strain is increased (see Figure 2). The results obtained by MD and DFT show similar behavior. The angle variation is larger in the MD atomistic simulations. Note that the fracture strain is slightly below 15% in MD, while it is around 19% in DFT [20].

For strains along the zigzag direction (Figure 3), the bond Z is elongated while the bond A is not. In MD the bond A is independent of strain, due to the fact that the force field contains solely first neighboring stretching terms [22], while in DFT the bond A shrinks. Regarding the strain variation of bond angles, angle α increases and ζ decreases, as expected. In the MD case, the decrease of graphene’s size in the lateral direction is exclusively due to the increase of angle α. The fracture strain in this direction of tension is around 28% in MD and around 24% in DFT [20].

Comparing our results with the MD data presented in reference [17] we observe similar trends, except for the case of A bonds in strains along the zigzag direction, where these bonds are found to increase with strain in that work. This behavior is different from what we obtain here, as our MD simulations show no variation of this bond length, while the DFT computations result in decreasing lengths of these bonds with strain. The bond length and bond angle deformations presented in Figure 5 of reference [22], using DFT calculations, are in good agreement with the results of this work, even at a quantitative level.

In order to facilitate efficient use of the bond length and bond angle deformations with the applied strain in potential strain engineering devices or other applications, we provide analytical relations describing these dependencies, which are obtained through appropriate fittings of the numerical results presented in Figure 2 and Figure 3. In particular, we observed that quadratic functions are able to accurately fit the numerical data shown above.

Thus, the lengths of both types of bonds (A and Z) are changing with the strain according to the relation

(1) $$L=L_{0}+C_{1}\;\varepsilon +C_{2}\;\varepsilon^{2},$$

where L is the bond length in Å at strain ε, $L_{0}$ is the bond length in the absence of strain (which is equal to 1.42 Å in MD, 1.424 Å in DFT with GGA/PBE functionals, and 1.412 Å in DFT with LDA/PZ functionals), $C_{1}$ and $C_{2}$ are coefficients (shown in Table 1 and Table 2 for strains applied along the armchair and the zigzag directions, respectively), and ε is the percentage strain.

Similarly, the strain dependence of both types of bond angles, α and ζ, is described by the formula

(2) $$\gamma =120^{{}^{\circ}}+\Gamma_{1}\;\varepsilon +\Gamma_{2}\;\varepsilon^{2},$$

where γ is the bond angle in degrees at strain ε, $\Gamma_{1}$ and $\Gamma_{2}$ are coefficients (shown in Table 3 and Table 4 for strains applied along the armchair and the zigzag directions, respectively), and ε is the percentage strain.

A very crude approximation of the A bond length variation with the strain along the armchair direction can be obtained through the analytical derivation of the extension of a single bond, described by the stretching potential used in our MD calculations, under an applied force. In particular, the Morse potential used for the carbon-carbon bond stretching in graphene is $V=D\;{\left[\exp\left(-a\left(r-r_{0}\right)\right)-1\right]}^{2}$, where D  =5.7 eV, a  =1.96 Å^−1^, and $r_{0}\;=$1.42 Å is the equilibrium distance [20]. Applying a constant tensile force F at the ends of such a bond, one can easily obtain the force extension relationship. Then taking into account that $F=\sigma \;\left(\sqrt{3}/2\right)\;r_{0}$, where σ is the corresponding stress and $\left(\sqrt{3}/2\right)\;r_{0}$ is the distance at which tensile forces are applied on the atoms of a zigzag edge in graphene, and by substituting the stress–strain relation $\sigma =\varepsilon \;E_{2D}$ with $E_{2D}=$320 N/m [20], one finally obtains that the extension of an A bond is

$$r=r_{0}-(1/a)\;\;\ln\left\{0.5\left[1+\sqrt{1-\varepsilon \sqrt{3}r_{0}E_{2D}/Da}\right]\right\}.$$

This analytical formula deviates by a factor of 2 from the numerical results of the strain dependence of the A bond length shown in Figure 2a. In the linear regime, for relatively small strains, the last formula gives $r=r_{0}+\varepsilon \sqrt{3}r_{0}E_{2D}/4Da^{2}$, where by substituting the values of the parameters mentioned above, we obtain the coefficient multiplying the strain as exactly half of the value of $C_{1}$, which is given in Table 1 for the A bond.

### 3.2. Optical Phonons of Uniaxially Strained Graphene at the Center of the Brillouin Zone

**Figure 4:**
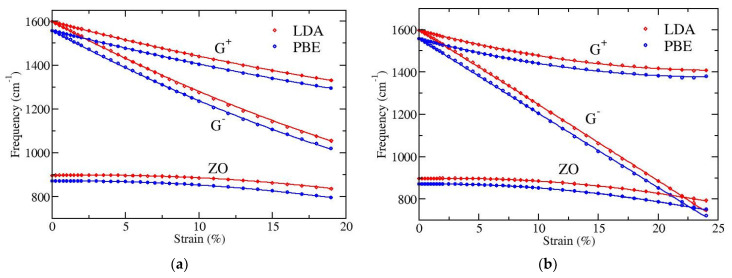
Strain dependence of the in-plane optical phonons (G band), that splits into G**^+^** and G**^–^** bands, and of the out-of-plane optical mode ZO for strain in the (**a**) armchair and (**b**) zig-zag directions, as obtained by DFT calculations at the level of LDA/PZ (red) and GGA/PBE (blue) functionals.

We have performed DFT calculations in order to investigate the dependence on strain of the long wavelength optical phonons at the Γ point and to quantify the splitting of the doubly degenerate E_2g_ mode (G band). LDA and PBE functionals have again been used, as previously. The results are presented in Figure 4. Both functionals show the same behavior, apart from a small (almost constant) shift of the optical frequencies; the PBE functional gives a few percent (2–3%) lower frequencies than LDA. In particular, for zero strain the doubly degenerate LO/TO optical frequency at Γ point is found 1598 cm^−1^ with LDA and 1557 cm^−1^ with PBE, while the out-of-plane ZO optical mode is at 896 cm^−1^ and 871 cm^−1^, respectively.

Figure 4a depicts results for strain applied along the armchair direction of graphene and Figure 4b for strain along the zigzag direction. All displayed phonons show softening, i.e., their frequencies decrease with increasing strain. The G**^−^** band exhibits the larger softening and the ZO mode the smaller one. The softening of the G**^+^** and G**^−^** bands has been experimentally observed [44,45] because these modes are Raman active. G**^−^** corresponds to an eigenmode parallel to the direction of strain, thus bearing higher extension and showing larger softening, while G**^+^** corresponds to a perpendicular to strain eigenmode. Note that the strong softening of the G**^−^** mode leads to a crossing with the lower frequency out-of-plane ZO mode when the tension is along the zigzag direction of graphene, where larger strains (more than 20%) can be applied (see Figure 4b).

To quantify the strain dependence of the optical modes at the center of the Brillouin zone, we have fitted the DFT obtained numerical data with the quadratic formula

(3) $$\omega =\omega_{0}+\omega_{1}\;\varepsilon +\omega_{2}\;\varepsilon^{2},$$

where ω is the frequency of the corresponding mode in cm^−1^, $\omega_{0}$ is the frequency at zero strain, and $\omega_{1}$ and $\omega_{2}$ are linear and second order coefficients, respectively, on the percentage strain ε. The results of these fittings are presented in Table 5 and Table 6 for the cases where the strain is along the armchair (Figure 4a) and the zigzag (Figure 4b) directions, respectively.

As already noted, the G**^+^** and G**^−^** bands are Raman active and their softening rates have been experimentally determined. A detailed discussion in reference [45] was able to isolate the effect of the substrate on the measured G**^+^** and G**^–^** softening, leading to corresponding redshift rate estimates for free standing graphene which are around −18 and −36 cm^−1^/(%strain) for G**^+^** and G**^−^**, respectively, for strains up to around 1%. For such a small strain the softening rates are provided directly by the coefficient $\omega_{1}$ of Equation (3). The values of $\omega_{1}$ presented in Table 5 and Table 6 for the G**^+^** and G**^−^** bands are in agreement with these experimental observations.

**Figure 5:**
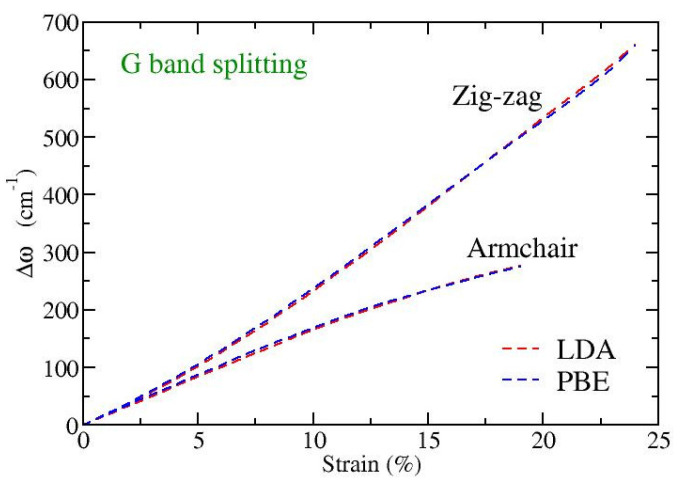
Strain dependence of the G-band splitting, $\Delta \omega =\omega^{+}-\omega^{-}$, for strains along the armchair and zigzag directions, as obtained by DFT calculations at the level of LDA/PZ (red) and GGA/PBE (blue) functionals.

Finally, we have explicitly calculated the variation with strain of the frequency splitting, $\Delta \omega =\omega^{+}-\;\;\omega^{-}$, between the perpendicular (G**^+^**) and parallel to strain (G**^−^**) in-plane eigenmodes, with $\omega^{+}$ and $\omega^{-}$ being their corresponding frequencies. In Figure 5 this dependence for strains applied along the armchair or the zigzag directions is shown. We see that while for small strains, up to 1–2%, the G-band splitting is independent of the direction of strain, for larger strains there is a strong dependence on the direction of the applied tension. The higher the strain, the larger this difference, where a tension along the zigzag direction exhibits larger G-band splitting than a similar strain in the armchair direction. Note that experimental observations in different directions of strain, as well as theoretical calculations, have found isotropic behavior regarding the G**^+^** and G**^−^** softening rates and the resulting G-band splitting [45]. However, both the experimental data and the theoretical computations were restricted in small strains (up to around 1%) in that work [45]. Our results shown in Figure 5 are consistent with these reports; for such small values of strain an isotropic behavior of the splitting is revealed. However, this behavior is no longer valid for larger strains.

A linear fitting of the splittings depicted in Figure 5 at small strains (below 1%) results in a rate of increase of the splitting with the strain around 17–18 cm^−1^/%strain, in both directions. This is in very good quantitative agreement with the experimental and theoretical results discussed in page 4 and page 5, respectively, of reference [45]. A crude analytical estimate of the splitting through the second derivative of the Morse stretching potential (see the discussion at the end of Section 3.1), when one substitutes the MD derived A and Z bond-length extensions on each direction of strain, leads to splitting around 30–35 cm^−1^/%strain, which are about twice the above mentioned DFT obtained values.

## 4. Conclusions

We discuss the strain dependence of the structural characteristics (bond lengths and bond angles) as well as of the long wavelength optical phonons (the G**^+^** and G**^−^** in-plane modes and the ZO out-of-plane mode) of graphene. Strains along the high-symmetry armchair and zigzag directions are considered.

The variation with strain of the lengths of the two distinct types of bonds (A and Z) and the two distinct types of angles (α and ζ) is obtained through density functional theory, using two different functionals (LDA/PZ and GGA/PBE), and by molecular dynamics atomistic simulations. In all these cases, the numerical data of the strain dependence of graphene’s geometrical parameters (bond lengths and bond angles) are fitted to appropriate quadratic analytical formulae.

Density functional theory computations with the functionals LDA/PZ and GGA/PBE were also used to determine the strain response of the optical phonons at the center of the Brillouin zone. A softening of the three optical phonons was observed, which is stronger for the G**^−^** band that corresponds to in-plane eigenmode parallel to the direction of strain. Through fitting, analytical relations are presented providing the strain dependence of the optical mode frequencies. The G-band splitting and its variation with strain was also calculated. For small strains, this splitting is isotropic, while for larger strains there is a strong dependence on the direction of tension. In the latter case, larger splitting appears for strains along the zigzag direction. Our results are consistent at a quantitative level with existing experimental observations as well as other theoretical data for small strains up to 1%.

## Figures and Tables

**Table 1:** Coefficients $C_{1}$ [in Å/(%strain)] and $C_{2}$ [in Å/(%strain)^2^] of the strain dependence of the lengths [see Equation (1)] for the bond types A and Z, when the strain is applied along the armchair direction, as obtained by the results of the different theoretical methods used in this work.

Bond Type	Calculation Method	$C_{1}$	$C_{2}$
**A**	DFT - GGA/PBE	0.011	0.00035
**A**	DFT - LDA/PZ	0.010	0.00034
**A**	MD	0.011	0.00024
**Z**	DFT - GGA/PBE	0.0035	−0.00013
**Z**	DFT - LDA/PZ	0.0035	−0.00012
**Z**	MD	0.0031	−0.000046

**Table 2:** Coefficients $C_{1}$ [in Å/(%strain)] and $C_{2}$ [in Å/(%strain)^2^] of the strain dependence of the lengths [see Equation (1)] for the bond types A and Z, when the strain is applied along the zigzag direction, as obtained by the results of the different theoretical methods used in this work.

Bond Type	Calculation Method	$C_{1}$	$C_{2}$
**A**	DFT - GGA/PBE	−0.000060	−0.000089
**A**	DFT - LDA/PZ	0.000082	−0.000083
**A**	MD	0	0
**Z**	DFT - GGA/PBE	0.0090	0.000081
**Z**	DFT - LDA/PZ	0.0087	0.000084
**Z**	MD	0.0088	0.000080

**Table 3:** Coefficients $\Gamma_{1}$ [in deg/(%strain)] and $\Gamma_{2}$ [in deg/(%strain)^2^] of the strain dependence of the angles [see Equation (2)] for the bond angles of type α and ζ, when the strain is applied along the armchair direction, as obtained by the results of the different theoretical methods used in this work.

Angle Type	Calculation Method	$\Gamma_{1}$	$\Gamma_{2}$
**α**	DFT - GGA/PBE	−0.80	0.027
**α**	DFT - LDA/PZ	−0.82	0.026
**α**	MD	−0.83	0.020
**ζ**	DFT - GGA/PBE	0.40	−0.014
**ζ**	DFT - LDA/PZ	0.41	−0.013
**ζ**	MD	0.41	−0.010

**Table 4:** Coefficients $\Gamma_{1}$ [in deg/(%strain)] and $\Gamma_{2}$ [in deg/(%strain)^2^] of the strain dependence of the angles [see Equation (2)] for the bond angles of type α and ζ, when the strain is applied along the zigzag direction, as obtained by the results of the different theoretical methods used in this work.

Angle Type	Calculation Method	$\Gamma_{1}$	$\Gamma_{2}$
**α**	DFT - GGA/PBE	0.74	−0.014
**α**	DFT - LDA/PZ	0.77	−0.014
**α**	MD	0.80	− 0.013
**ζ**	DFT - GGA/PBE	−0.37	0.0069
**ζ**	DFT - LDA/PZ	−0.39	0.0068
**ζ**	MD	−0.40	0.0064

**Table 5:** Zero strain values $\omega_{0}$ (in cm^−1^), and coefficients $\omega_{1}$ [in cm^−1^/(%strain)] and $\omega_{2}$ [in cm^−1^/(%strain)^2^] of the strain dependence [see Equation (3)] of the optical in-plane (G**^+^** and G**^−^**) and out-of-plain ZO phonons at the Γ point, when the strain is applied along the armchair direction, as obtained by DFT calculations.

Optical Mode	Calculation Method	$\omega_{0}$	$\omega_{1}$	$\omega_{2}$
**G^+^**	GGA/PBE	1557	−16.94	0.16
**G^+^**	LDA/PZ	1598	−17.53	0.18
**G^−^**	GGA/PBE	1557	−35.86	0.39
**G^−^**	LDA/PZ	1598	−35.53	0.35
**ZO**	GGA/PBE	871	0.58	–0.24
**ZO**	LDA/PZ	896	1.29	–0.23

**Table 6:** Zero strain values $\omega_{0}$ (in cm^−1^), and coefficients $\omega_{1}$ [in cm^−1^/(%strain)] and $\omega_{2}$ [in cm^−1^/(%strain)^2^] of the strain dependence [see Equation (3)] of the optical in-plane (G**^+^** and G^−^) and out-of-plain ZO phonons at the Γ point, when the strain is applied along the zigzag direction, as obtained by DFT calculations.

Optical Mode	Calculation Method	$\omega_{0}$	$\omega_{1}$	$\omega_{2}$
**G^+^**	GGA/PBE	1557	−14.66	0.30
**G^+^**	LDA/PZ	1598	−15.10	0.30
**G^−^**	GGA/PBE	1557	−35.40	0.012
**G^−^**	LDA/PZ	1598	−34.90	−0.035
**ZO**	GGA/PBE	871	0.41	−0.23
**ZO**	LDA/PZ	896	1.14	−0.23

## Data Availability

The data of this study are available from the corresponding author upon reasonable request.
